# Effects of channel size, wall wettability, and electric field strength on ion removal from water in nanochannels

**DOI:** 10.1038/s41598-021-04620-x

**Published:** 2022-01-12

**Authors:** Filippos Sofos, Theodoros E. Karakasidis, Ioannis E. Sarris

**Affiliations:** 1 Condensed Matter Physics Laboratory, Physics Department, University of Thessaly, 35100 Lamia, Greece; 2 Department of Mechanical Engineering, University of West Attica, 12244 Athens, Greece

**Keywords:** Nanoscale materials, Theory and computation, Statistical physics

## Abstract

Molecular dynamics simulations are employed to estimate the effect of nanopore size, wall wettability, and the external field strength on successful ion removal from water solutions. It is demonstrated that the presence of ions, along with the additive effect of an external electric field, constitute a multivariate environment that affect fluidic interactions and facilitate, or block, ion drift to the walls. The potential energy is calculated across every channel case investigated, indicating possible ion localization, while electric field lines are presented, to reveal ion routing throughout the channel. The electric field strength is the dominant ion separation factor, while wall wettability strength, which characterizes if the walls are hydrophobic or hydrophilic has not been found to affect ion movement significantly at the scale studied here. Moreover, the diffusion coefficient values along the three dimensions are reported. Diffusion coefficients have shown a decreasing tendency as the external electric field increases, and do not seem to be affected by the degree of wall wettability at the scale investigated here.

## Introduction

Controlling ion transport in aqueous solutions is of paramount importance for applications related to water desalination, batteries, and cellular membranes^1^, energy from salt gradient^2^ and nanopore sensing^3^. Current experiments on the field have suggested novel materials suitable for water/ion applications, such as graphene and graphene oxide (GO), carbon nanotubes (CNTs), metal–organic frameworks (MOFs), and membrane protein channels in block copolymers (MP-BCPs)^4–8^. All these experimental efforts have been incorporated to address the insightful research suggested by simulations at this scale. The effects of confinement have been experimentally understood in flows inside Carbon Nanotubes (CNTs)^9,10^, while, recently, manufacturing advances have made it possible to experiment over water flow among planar surfaces^11^.

The concept of removing unwanted substances from water through a nanoscale membrane, where ions are blocked by a permeable structure, has been widely studied in the literature^12–14^. Membrane-based processes, such as the Reverse Osmosis (RO) and nanofiltration (NF), have been established as low-cost, effective solution, replacing classical distillation methods^15^. In electric-assisted desalination, ions are extracted from the saline solution with aid of an electric field. Current methods being exploited include electrodialysis (ED), capacitive deionization (CDI), and flow-electrode capacitive deionization (FCDI). Special emphasis is given to FCDI, in which, continuous desalination is applicable (see more details in a recent review^16^).

Nanometer-sized channels fit in such applications due to their close proximity to relevant biological processes. The size of nanochannels can facilitate or, on the other hand, block ion transport^17^. Another significant parameter is the application of an external electric field, *E*, with direction parallel or perpendicular to the flow^18,19^. By controlling the external electric field, an atomic-scale ion transistor is created, ensuring ultrafast and selective ion transport, mimicking biological nanochannels with ON/OFF capability^20^. In every case, special care needs to be taken not only regarding water sensitivity under an electric field^21^, but, also, confinement effects. The multiple-interaction environment inside a charged nanochannel is governed both by electrostatic and frictional interactions of fluid particles with confining surfaces^22^.

In addition, at the nanoscale, the pivotal role of wall wettability has to be underlined, as it affects most static and dynamic fluid properties^23–25^. It has been shown that by tuning the wall wettability, it is possible to generate net unidirectional flow^26^. Hydrophilic/hydrophobic surfaces have been found to offer a promising means for controlling water flow. Carbon nanotubes (CNTs) with modified hydrophilic-hydrophobic tips have been incorporated to achieve fast water transport and ion selectivity^27^. In this way, ions are difficult to enter the tube due to high energy barriers at the tips, making the CNT a potential water filter. Moreover, in the presence of an external electric field, water molecule contact angle on the surface, which characterizes wall hydrophobicity and hydrophilicity, is affected^28^. Inside biological or synthetic nanopores, it has been also found that the pore hydration probability increases when the applied electric field increases, too^29^.

From a simulation perspective, classical molecular dynamics simulations have been incorporated for the investigation of such systems, providing close-to-reality approaches of internal processes, assisting experimental procedures, and pointing key theoretical issues which are vital for the establishment of functional models^30,31^. For example, water properties, such as the radial distribution function, dipole orientation, or, the number of hydrogen bonds, to mention a few, are subject to change. Under a static electric field between 0.01 and 0.2 V/Å, the strength of hydrogen bonds increases and results in diffusion coefficient anisotropy^32^. As a result, anisotropy is also induced in water viscosity, where the component parallel to *E* increases and the perpendicular component decreases, as *E* increases monotonically^33^. Continuum approaches on ionic transport include the Poisson-Nerst-Planck (PNP) theory, which can be less computationally demanding than MD, but, on the other hand, it succeeds only at the bulk and fails to approach regions near the channel walls^34–36^.

In this work, MD simulations have been performed to develop and refine a nanochannel desalination model, in which ion transport towards the wall is facilitated under the effect of an external electric field, perpendicular to the walls^37,38^. Emphasis has been given on the investigation of the underlying mechanism that controls ion and water molecules behavior, with aid of a large number of independent MD simulations. Numerical calculations of the potential energy and the electric field inside the nanochannel have been performed, while fluid diffusion coefficients have been extracted along the three dimensions. The main parameters that have been found to affect ion drift inside the channel (ion concentration close to seawater and less) are the channel width, spanning over the range *h* = 3–21 nm, the external electric field, for values *E* = 0.0–10.0 V/nm, and, up to a certain degree in small nanochannels , the wall/fluid interaction strength as $0.1 < \varepsilon_{wf} /\varepsilon_{ff} < 1.0$ .

## Methods

### Model and implementation details

**Figure 1:**
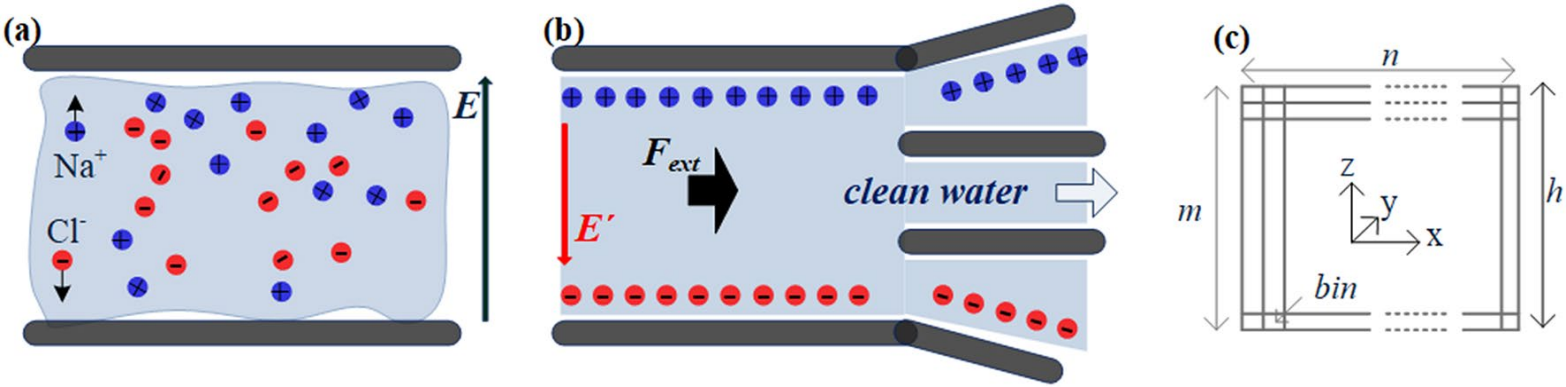
Ion/water flow between two solid walls, channel model (periodic in *x-* and *y-* dimensions, while, *h*, is the channel height, in the range $3\;{\text{nm}} \le h \le 62\;{\text{nm}}$). Blue and red circles are Na^+^ and Cl^−^ ions, respectively*.* The external electric field *E*_*z*_ is applied perpendicular to the flow (driven by the external force *F*_*x*_). Here is the channel: (**a**) in the beginning of the simulation, (**b**) after the application of *E*, where an internal *E´* is created, while *F*_*x*_ drives the flow, (**c**) the division in computational bins *m* $\times$ *n* $\times$ *k* (*k* is in the *y*-dimension).

Molecular dynamics simulations of Na^+^, Cl^−^, and water solution, in various concentrations, confined between two impermeable, solid walls are performed (Fig. 1a). For the ease of simulation, the system is considered periodic at the *x-*, *y-* and fixed at the *z-*direction. Details on materials, parameters, and dimensions incorporated are given in the Supplementary Material.

The SPC/E water model, which has been found to reproduce adequately the structural and dynamic properties of water^39^, is incorporated, composed of Lennard–Jones (LJ) and Coulombic potential terms for the solution. A particle–particle particle-mesh (PPPM) method has been used to calculate the long-range electrostatic force^40^. The LJ potential between two particles *i* and *j* is described by the equation

1 $$u\left( {r_{ij} } \right) = \left\{ \begin{array}{ll} 4\varepsilon \left[ {\left( {\frac{\sigma }{{r_{ij} }}} \right)^{12} - \left( {\frac{\sigma }{{r_{ij} }}} \right)^{6} } \right]&\quad r_{ij} < r_{c} \hfill \\ 0&\quad\;r_{ij} \ge r_{c} \hfill \\ \end{array} \right.$$

where parameter *ε* indicates the interaction strength, *σ* defines the length scale, and *r*_*c*_ = 9 Å the cut-off radius. Moreover, coulombic interaction between hydrogen, oxygen, natrium and chloride atoms is given by ${V}_{c}=\frac{C{q}_{i}{q}_{j}}{{\varepsilon }_{0}{r}_{ij}}$, where *C* is the energy conversion constant, *q*_*i*_ and *q*_*j*_ the charges of interacting atoms, and *ε*_*0*_ is the dielectric constant.

Τhe ratio of wall-to-fluid interaction $\varepsilon_{wf} /\varepsilon_{ff}$ controls wall wettability and is inversely analog to the value of the contact angle between the surface and the fluid^41–43^. The wall is treated as strong hydrophobic for $\varepsilon_{wf} /\varepsilon_{ff}$ = 0.1 (large contact angle), hydrophobic for $\varepsilon_{wf} /\varepsilon_{ff}$ = 0.2, hydrophilic for $\varepsilon_{wf} /\varepsilon_{ff}$ = 0.5 and strong hydrophilic for $\varepsilon_{wf} /\varepsilon_{ff}$ = 1.0 (near-zero contact angle) . Wall particles are initially arranged on a face-centered cubic (fcc-101) lattice, which is common practice in MD, LJ simulations^38^.

The application of an external electric field (acting analog to a solution between two charged surfaces), ideally results in anion drift towards the “positive” and cation towards the “negative” surface (Fig. 1b). The electric field originates from the homogeneous distribution of opposite sign charges on the two walls, resulting in an electric force *F*_*e*_ = *qE*_*z*_ acting on the *z*-dimension. The applied *E*_z_ in this work is investigated over the range 0.0–10.0 V/nm. It has been argued that, when *E* > 3.5 V/nm, ab-initio simulations revealed that the dissociation of a water molecule occurs^44^. The water molecules are prone to undergo autoionization to form H3O^+^ and OH^−^ ions^45^*.* Nevertheless, for the theoretical model presented here, water bonds are constrained using the SHAKE algorithm^40^ and no dissociation phenomena are observed. Furthermore, it has been shown that the application of an electric field can cause phase change (water freezing). However, when the field applies perpendicular to the wall levels (as in our case, in our value range), no freezing effect occurs^46^.

The proposed desalination procedure succeeds when most ions approach the walls, under the effect of *E*_*z*_. As ions are forced to drift away from the solution, clean water remains in the channel interior and has to be appropriately gathered on the outlet (not shown here). Wall atoms absorb the increase in fluid kinetic energy due to the application of the external fields, and, to maintain constant system temperature (*T* = 300 K), Nosé–Hoover thermostats are employed at the walls.

In order to perform realistic MD simulations, system properties have to be clearly defined. As our investigation goes over various channels of different heights, properties like temperature, pressure and ion concentration have to be the same so that comparisons are made under the same conditions across all channel heights. Initial system setup is of primary importance. A common establishment is the addition of reservoirs before and after the nanochannel to ensure bulk conditions^47^. Another, challenging approach is based on chemical potential calculations^48^, while a more convenient one includes an initial mechanical pressure application to achieve the desired system properties^49^. By selecting one of these methods, one can be sure that no nanobubbles or depletion layers are formed at the walls and alter the results.

In our system, the simulation procedure in LAMMPS follows these steps: (I) Fluid and wall atoms are placed inside the simulation box with fcc structure with parameters that correspond to the desired density. The two non-periodic box dimensions, *L*_*x*_ and *L*_*y*_, are controlled by a barostat and left to equilibrate under a NPT scheme for 1 ns to ensure constant pressure and temperature, and fluid particles are left to attain their positions and velocities in the box. Wall particles remain frozen. (II) After the simulation box attains its final dimensions, an energy minimization stage follows to remove artificially imposed strains in the initial configuration and the simulation system evolves under a NVE scheme for another 1 ns. (III) An intermediate step with the electric field applied follows and temperature is controlled under a canonical (NVT) ensemble by Nosé–Hoover thermostats, for 5 ns. (V) Consecutive NVT production runs are made with the electric field on, where we obtain samples from atomic positions, velocities etc. to calculate properties under an electric field. For statistical reasons, we have considered 4 consecutive, independent runs of 5 ns each and averaged the extracted properties. Simulation timestep is *Δt* = 1 fs.

### Computational details

The effect of the application of an external electric field on ion/water flows in conduits is investigated. Taking further into consideration the hydrophobic/hydrophilic nature of the wall and the repulsive/attractive forces between positive and negative ions, a multivariate interaction environment is considered. For this reason, potential energy plots are extracted in order to locate regions of low energy where ions could be trapped and affect system dynamics. If these regions lay far from the walls, this would be evidence of poor desalination performance. Since the system is periodic in *x*- and *y*-directions, it would be of interest to calculate the potential energy as function of *z*.

Potential energy is given by

2 $$U = U_{LJ} + V_{c}^{ } + U_{E}^{ }$$

The term $U_{LJ}$ is the local Lennard–Jones interaction potential and encompasses wall/fluid interactions in terms of hydro/ion-phobicity and hydro/ion-philicity. The second term, $V_{c}^{{}}$, is the potential energy associated with long-range electrical interactions between all charged atoms, H^+^ and O^−^ from water molecules, and Na^+^, Cl^−^ as free-running ions in the solution. The outcome of the application of the external field applied perpendicular to the walls, *E*_*z*_, is associated with ${U}_{E}$ .

Equation 2 has been incorporated to extract the potential energy map inside the channels. As the aim of the proposed model is to present a method of ion drift towards the walls, this would be a valuable information, since low (negative) potential regions can reveal possible ion trapping positions inside the channel that put a barrier on their transport.

The induced electric field due to the presence of a number of *N*_*c*_ charged atoms inside a bin is

3 $$\vec{E}_{ion} \left( {\vec{r}_{i} } \right) = - \left. {\frac{{\partial V^{c} }}{\partial x}} \right|_{{\overrightarrow {{r_{i} }} }} {\vec{\text{x}}} - \left. {\frac{{\partial V^{c} }}{\partial y}} \right|_{{\overrightarrow {{r_{i} }} }} {\vec{\text{y}}} - \left. {\frac{{\partial V^{c} }}{\partial z}} \right|_{{\overrightarrow {{r_{i} }} }} {\vec{\text{z}}}$$

or equivalently

4 $$\vec{E}_{ion} \left( {\vec{r}_{i} } \right) = \frac{1}{{4\pi \varepsilon_{0} \varepsilon_{r} }}\mathop \sum \limits_{j = 1,j \ne i}^{{N_{c} }} \frac{{q_{j} }}{{\left| {\vec{r}_{ i} - \vec{r}_{j} } \right|^{3} }}\left( {\vec{r}_{ i} - \vec{r}_{j} } \right)$$

where ${\varepsilon }_{r}$ the dielectric constant of water. By adding the external field $\vec{E}_{ext}$, distributed equally throughout the channel, we obtain the local value $\vec{E}_{ }^{ }$ as

5 $$\vec{E} = \vec{E}_{ion}^{ } + \vec{E}_{ext}$$

The average diffusion coefficient throughout the channel is obtained from the time-averaging of the mean square displacement of *N* fluid particles, using the Einstein’s relation, as

6 $$MSD = \frac{1}{N}\left\langle {\mathop \sum \limits_{j = 1}^{N} \left[ {{\varvec{r}}_{j} \left( t \right) - {\varvec{r}}_{j} \left( 0 \right)} \right]^{2} } \right\rangle$$

And

7 $$D = \mathop {lim}\limits_{t \to \infty } \frac{1}{6Nt}\left\langle {\mathop \sum \limits_{j = 1}^{N} \left[ {{\varvec{r}}_{j} \left( t \right) - {\varvec{r}}_{j} \left( 0 \right)} \right]^{2} } \right\rangle$$

where ${\varvec{r}}_{j}$ is the position vector of the *j*th atom. In each dimension (*x*, *y*, or *z*) we distinct the three components of the diffusion coefficient as

8 $$\begin{gathered} D_{x} = \mathop {lim}\limits_{t \to \infty } \frac{1}{2t}\frac{1}{N}\left\langle {\mathop \sum \limits_{j = 1}^{N} \left[ {x_{j} \left( t \right) - x_{j} \left( 0 \right)} \right]^{2} } \right\rangle \hfill \\ D_{y} = \mathop {lim}\limits_{t \to \infty } \frac{1}{2t}\frac{1}{N}\left\langle {\mathop \sum \limits_{j = 1}^{N} \left[ {y_{j} \left( t \right) - y_{j} \left( 0 \right)} \right]^{2} } \right\rangle \hfill \\ D_{z} = \mathop {lim}\limits_{t \to \infty } \frac{1}{2t}\frac{1}{N}\left\langle {\mathop \sum \limits_{j = 1}^{N} \left[ {z_{j} \left( t \right) - z_{j} \left( 0 \right)} \right]^{2} } \right\rangle \hfill \\ \end{gathered}$$

We also calculate the parallel component *D*_*||*_ from the two non-confined directions (*x* and *y*) as

9 $$D_{||} = \mathop {lim}\limits_{t \to \infty } \frac{1}{4t}\frac{1}{N}\left\langle {\mathop \sum \limits_{j = 1}^{N} \left[ {r_{j}^{xy} \left( t \right) - r_{j}^{xy} \left( 0 \right)} \right]^{2} } \right\rangle$$

where, ${r}_{j}^{xy}$ is the position vector in *x*- and *y*-directions.

Values for diffusion coefficients are extracted inside the nanochannels under the electric field effect, after the simulation has reached its steady state, where ions have obtained their positions near the walls. During production simulation runs, the MSD samples values are stored and reported diffusion coefficient values are averaged over several sampling time windows, in order to achieve statistical accuracy. Calculations have been also made under flow conditions, presenting no significant difference on *D* values^50^.

## Results

### Potential energy and ion localization

In continuous methods, such as the PNP theory, ions are considered as continuous charge distributions and their concentration profile is easy to achieve. Apart from the macroscale, PNP theory has been successfully applied in smaller scales^51^. However, in MD methods, particles are treated as discrete mass points and the representation of ions by their mean field properties would give an over simplistic representation due to the small number of ions compared to the water molecules^35^.

**Figure 2:**
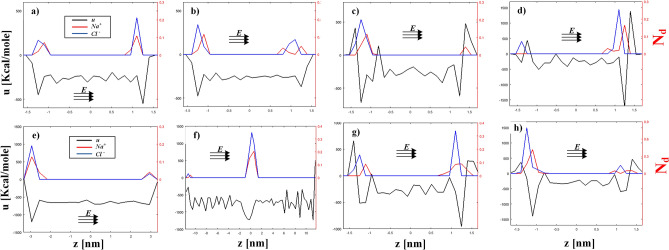
Potential energy for ion/water solution (*c* = 0.58 M, unless otherwise stated), in comparison with ion mass density, *N*_*d*_. Blue and red lines denote Cl^−^ and Na^+^ mass density, respectively, and refer to the right vertical axis. Black line is the calculated potential energy (left vertical axis). Potential values are averaged over the *xy*-directions. Simulation conditions are: (**a**) *E*_*z*_ = 0.001 V/nm, *h* = 3 nm, $\varepsilon_{wf} /\varepsilon_{ff } = 0.1$ (**b**) *E*_*z*_ = 0.1 V/nm, *h* = 3 nm, $\varepsilon_{wf} /\varepsilon_{ff } = 0.1$_,_ (**c**) *E*_*z*_ = 1.0 V/nm, *h* = 3 nm, $\varepsilon_{wf} /\varepsilon_{ff } = 0.1,$ (**d**) *E*_*z*_ = 1.0 V/nm, *h* = 3 nm, $\varepsilon_{wf} /\varepsilon_{ff } = 1.0$, (**e**) *E*_*z*_ = 0.1 V/nm, *h* = 6 nm, $\varepsilon_{wf} /\varepsilon_{ff } = 0.1$, (**f**) *E*_*z*_ = 0.1 V/nm, *h* = 21 nm, $\varepsilon_{wf} /\varepsilon_{ff } = 0.1$, (**g**) *E*_*z*_ = 0.1 V/nm, *h* = 3 nm, $\varepsilon_{wf} /\varepsilon_{ff } = 0.1$, *c* = 0.92 M, (**h**) *E*_*z*_ = 0.1 V/nm, *h* = 3 nm, $\varepsilon_{wf} /\varepsilon_{ff } = 0.1$, *c* = 1.84 M.

To emphasize the impact of potential energy in ion concentration across the nanochannels, next we present ion mass density and potential energy in double-axis plots. The effect of the electric field strength on ion localization is investigated first on the strongly hydrophobic ($\varepsilon_{wf} /\varepsilon_{ff } = 0.1$) *h* = 3 nm nanochannel. The potential energy in the z-direction is presented for *E*_*z*_ = 0.001–0.1 V/nm in Fig. 2a–b. Regions of lower, negative potential energy are local minima which fluid particles prefer to occupy^52^. Due to the hydrophobic walls, regions that could attract water molecules near the walls are expected to form^42^. From the density of mass, *N*_*d*_, it is shown that two Na^+^ and Cl^−^ peaks have been created near the upper wall, and two shorter Cl^−^ peak near the lower channel wall (Fig. 2a). Potential energy is strongly negative near the walls, and this is evidence that ions are practically trapped inside this region. On the other hand, water particles are strongly attracted near a hydrophobic wall, and, as ions tend to create a hydrogen bonding network around them, clusters of ions are drifted towards the walls.

Next, the external electric field is increased to *E*_*z*_ = 0.1 V/nm (Fig. 2b). Near the lower wall, the potential energy distribution has created a negative well, surrounded by two peaks of positive potential. Inside this well, almost all ions have been trapped. It is obvious that at this electric field value, ion drift to the walls is facilitated. To project this result to a future desalination procedure, as depicted in Fig. 1b, one would expect that positive ions would drift to the upper wall and negative ions to the lower wall. Nevertheless, it has been shown that Na^+^ and Cl^−^ ions have formed clusters and drift together towards the walls. We attribute this behavior to nanoscale effects, since it has not been predicted by relevant continuum theory^34^. Even so, ions can be removed from the solution since they have approached the walls.

Wall/fluid interaction is another important parameter affecting fluid particle localization, especially near the walls where this effect is maximized. Two representative cases are presented for *h* = 3 nm, *E*_*z*_ = 1.0 V/nm, for $\varepsilon_{wf } /\varepsilon_{ff } = 0.1 - 1.0$, in Fig. 2c–d. Near the lower wall, where Cl^−^ ions are expected to migrate, the mass density plot for Cl^−^ presents a high peak, indicating that all negative ions are localized in this region, which is a negative potential energy region. This layer of negative ions is followed by a layer of positive ions (Na^+^), which are kept together due to electrostatic attraction. Only a small portion of Na^+^ ions have drifted to the upper wall, where another, negative potential region has been created. However, the negative potential region at the lower wall comes as a more probable candidate position for ions, since conditions of ion trapping have occurred, as the negative potential well is further surrounded by two positive potential peaks. For the strongly hydrophilic channel, $\varepsilon_{wf} /\varepsilon_{ff } = 1.0$, in Fig. 2d, ion behavior is reversed. Only a small percentage of Cl^−^ ions are located near the lower wall, trapped by two alternating negative/positive peaks of potential energy. On the other side, a layer containing all Na^+^ ions is observed near the wall, inside a negative potential well. This high positive ion concentration attracts most of the negative Cl^−^ ions and prevents them from drifting to the lower wall. The effect of wall wettability on the potential energy distribution across the channel is prominent, but at least for the conditions investigated here, has shown no significant impact on ion removal from the channel interior.

The channel height effect on potential energy and ion density values is also studied here. In this work, ion/water flow simulations have been performed for channel dimensions ranging from $3\,\mathrm{ nm}\le h\le 21\, \mathrm{nm}$. For the *h* = 6 nm channel (Fig. 2e), a strong negative potential energy region is observed close to the lower wall and a smaller one at the upper wall. All ions have been gathered in these two regions. A multiple interaction environment is observed for the *h* = 21 nm channels (Fig. 2f). Potential energy fluctuates around a constant negative value, presenting only one minimum in the channel centerline. This is where all ions have been gathered. We attribute this behavior to the fact that the induced external *E*_*z*_ = 0.1 V/nm is not strong enough to overcome internal energy barriers and lead ions towards the walls, as it does for the narrower channels shown before. It is a fact that wider channels obtain more electric energy when the same *E*_*z*_ applies, however, ions have larger distance to travel and more possibilities to be blocked inside a low potential region, away from the walls.

We also report on potential energy calculations for different salt concentrations, *c*, for the *h* = 3 nm, *E*_*z*_ = 0.1 V/nm, ${\varepsilon }_{wf}/{\varepsilon }_{ff }=0.1$ case. By increasing salt concentration to *c* = 0.92 M (Fig. 2g), simulation conditions ensure ion drift near the walls and there is no significant difference compared to the respective *c* = 0.58 M case (Fig. 2b), apart from the stronger negative behavior of the potential energy. Some Cl^−^ ions are localized near the lower wall and they have also attracted a number of Na^+^ ions to a neighboring layer. On the upper wall, Na^+^ ions (smooth peak) have approached the wall, followed by a layer of Cl^−^ ions (sharp peak). For *c* = 1.84 M (Fig. 2h), there are two sharp ion peaks at the lower wall, as most ions have been drifted there. This is attributed to the strongly negative potential energy in this region. From cases shown here, the proposed model seems to apply in a wide range of salt concentration values, from *c* = 0.58–1.84 M, without the need to increase the applied electric field.

### Electric field orientation

It has been found that, in similar models, when the electric field is applied perpendicular to the walls^38^, water molecules are aligned, with hydrogen atoms pointing to the negative direction. In the absence of ions in the solution, the electric field lines would be in the form of straight lines, parallel along the *z*-dimension. In the presence of ions, transport along the *z*-axis (towards the upper or the lower wall) is expected to emerge, as long as *E*_*z*_ is strong enough to overcome interatomic interactions. There are cases where ion separation is not clear; Na-Cl ion pairs are formed on low *E*_*z*_ strength and could move together towards the walls^53^, or, in the worst-case scenario, wander around the channel centerline. Furthermore, Na^+^ and Cl^−^ ions have different mechanisms of transport and diffuse at different rates^54^. Larger ions (Cl^−^) are less binded to water molecules and are, thus, more prone to be driven by an electric field, in contrast to smaller Na^+^ ions^17^. Therefore, ion movement is, somewhat, complicated. As ions move towards the upper or the lower wall, a new electric field, *E*_*ion*_, opposite to *E*_*z*_, is created, acting competitively. To shed light on this mechanism, further investigation has to be made.

**Figure 3:**
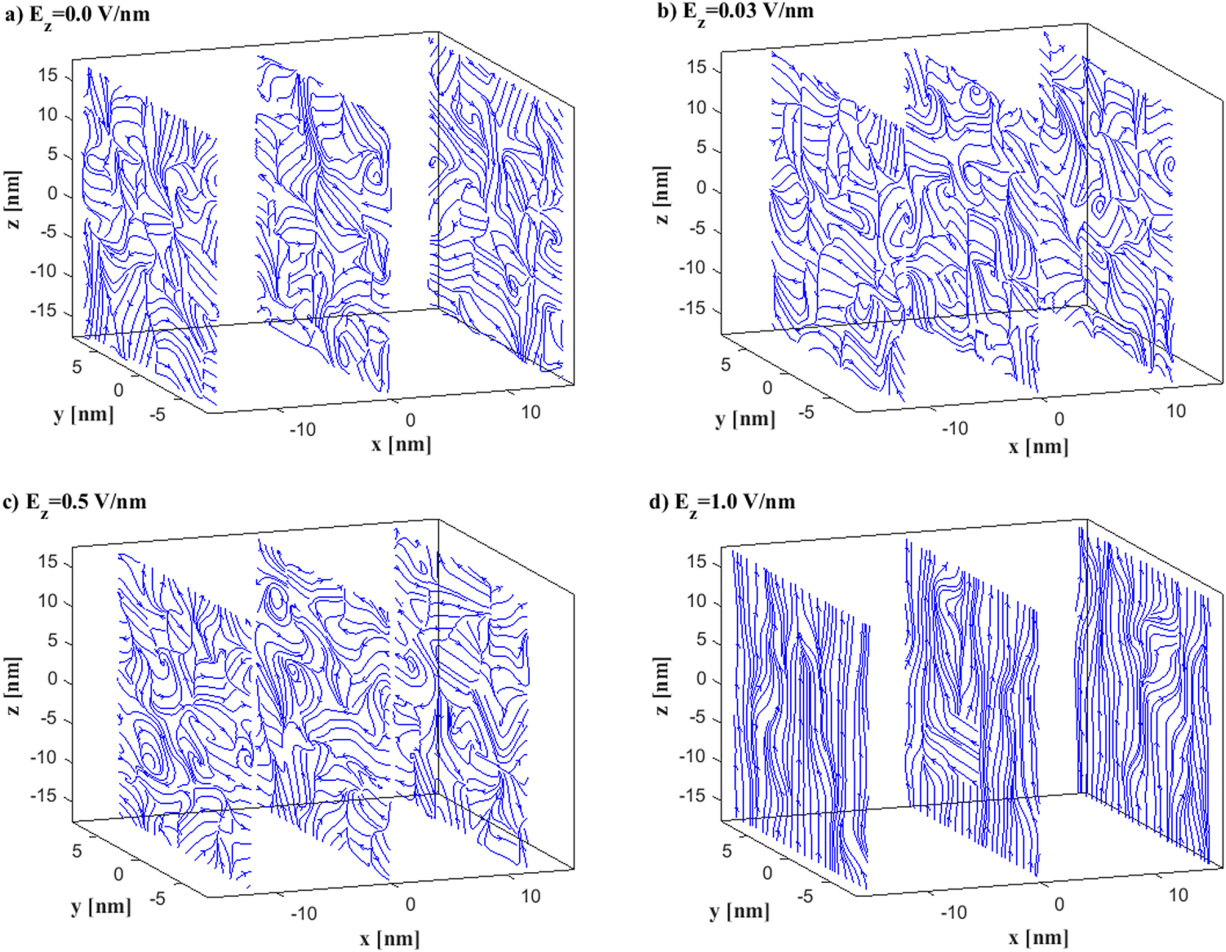
Electric field lines inside the *h* = 3 nm, $\varepsilon_{wf} /\varepsilon_{ff } = 0.1$ (**a**) *E*_*z*_ = 0.0 V/nm, (**b**) *E*_*z*_ = 0.03 V/nm, (**c**) *E*_*z*_ = 0.5 V/nm, (**d**) *E*_*z*_ = 1.0 V/nm. Three different *yz*-planes along the *x*-direction are shown for each case. Water/ions not shown for presentation reasons.

At first, we present the electric field distribution inside the *h* = 3 nm nanochannel case, ${\varepsilon }_{wf}/{\varepsilon }_{ff }=0.1$ (hydrophobic) and *E*_*z*_ = 0.0 V/nm. Three random slices across the *yz*-plane are shown in Fig. 3a. There is no clear direction of the lines. No significant effect is observed by increasing the electric field strength to *E*_*z*_ = 0.03 V/nm (Fig. 3b) and *E*_*z*_ = 0.5 V/nm (Fig. 3c). However, the electric field lines are becoming directional and follow the external electric field direction, *E*_z_, for *E*_*z*_ = 1.0 V/nm (Fig. 3d), although their direction is not uniform. This is evidence that ions are facilitated to drift towards the walls for *E*_*z*_ > 0.1 V/nm, unless other phenomena occur.

### Diffusion coefficients

As shown in Eqs. (6–9), the diffusion coefficients depend on the mean square displacement calculations, which may be a non-trivial task in terms of statistical accuracy. To reduce statistical errors, we performed for every case investigated here four independent simulations, each one of duration *t* = 5 ns (total duration 20 ns), where MSD-*x,y*,*z* values were saved every 10 timesteps (*Δt* = 1 fs).

**Figure 4:**
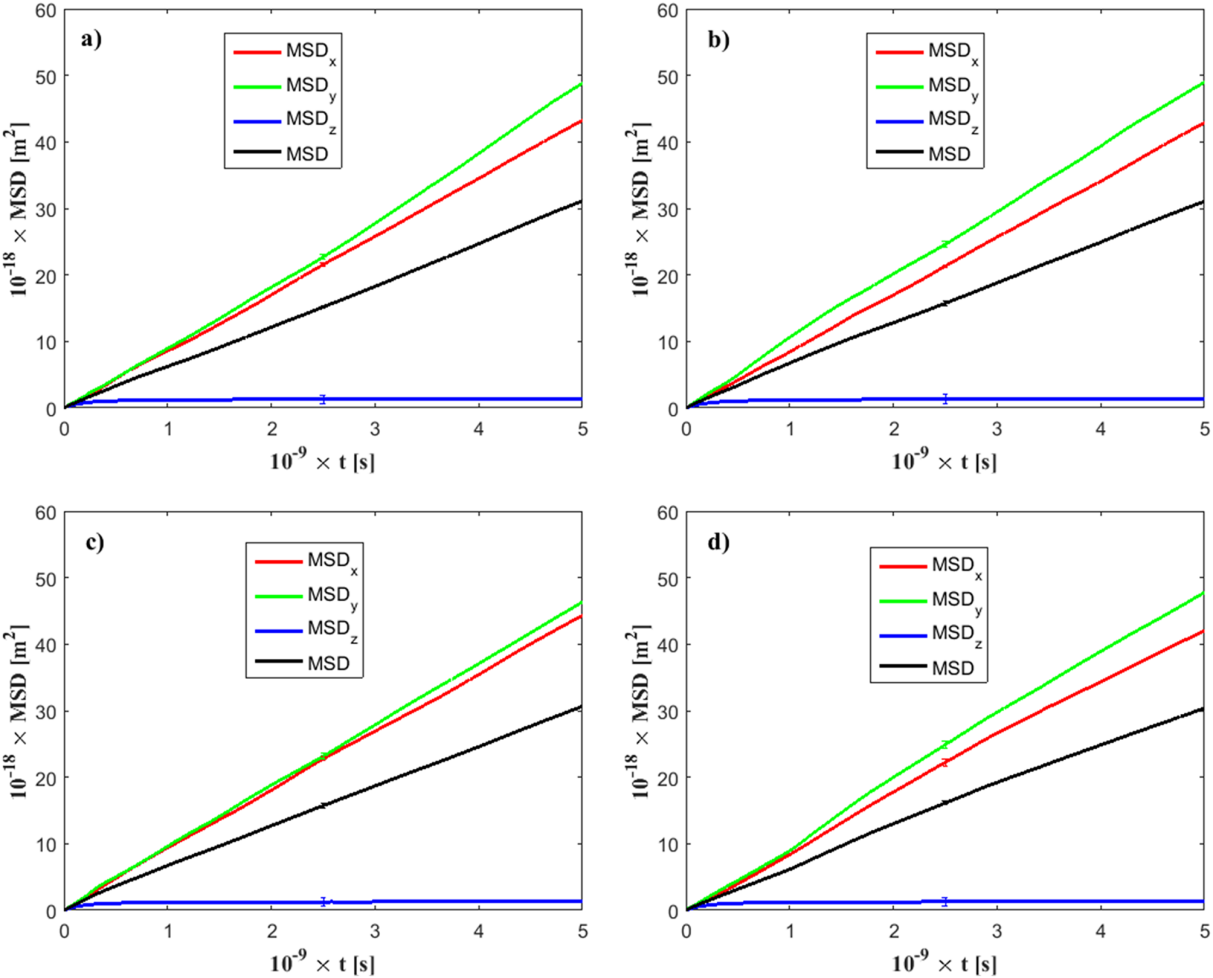
Calculated mean square displacement of fluid particles. Standard deviation is displayed in the middle of each line. *h* = 3 nm, $\varepsilon_{wf} /\varepsilon_{ff } = 0.1$. (**a**) *E*_*z*_ = 0.0 V/nm and no ions are present in the solution (pure water), (**b**) *E*_*z*_ = 1.0 V/nm and no ions are present in the solution, (**c**) *E*_*z*_ = 0.01 V/nm, for ion concentration *c* = 0.58 M, (**d**) *E*_*z*_ = 0.1 V/nm, for ion concentration *c* = 0.58 M.

Figure 4 presents the calculated MSD for various simulation cases. For means of comparison, simulation of pure water (no ions), no external electric field, and ${\varepsilon }_{wf}/{\varepsilon }_{ff }=0.1$ is shown in Fig. 4a. Even in the absence of ions and external electric forces, there exists pronounced anisotropy between the MSD components. For this channel case, confinement effects are strong, and impose strong anisotropy to the MSD_z_ component (perpendicular to the wall level). This effect is only due to the channel width. Slight anisotropy between the *x*- and *y*-component is also observed, due to statistical errors. The standard deviation is also plotted on each MSD component in Fig. 4. For the case where an external field *E*_*z*_ = 1.0 V/nm applies, in Fig. 4b, no significant change has been observed in terms of the calculated MSD channel value. However, the anisotropy between the *x*- and *y*-components has increased. For a solution of *c* = 0.58 M and a weak field of *E*_*z*_ = 0.01 V/nm (Fig. 4c), the MSD has been slightly decreased because of the addition of ions. Practically, no anisotropy exists between the MSD_x_ and MSD_y_ components. On the opposite, for a stronger field *E*_*z*_ = 0.1 V/nm (Fig. 4d), MSD_x_ and MSD_y_ components have shown slight anisotropic behavior.

**Figure 5:**
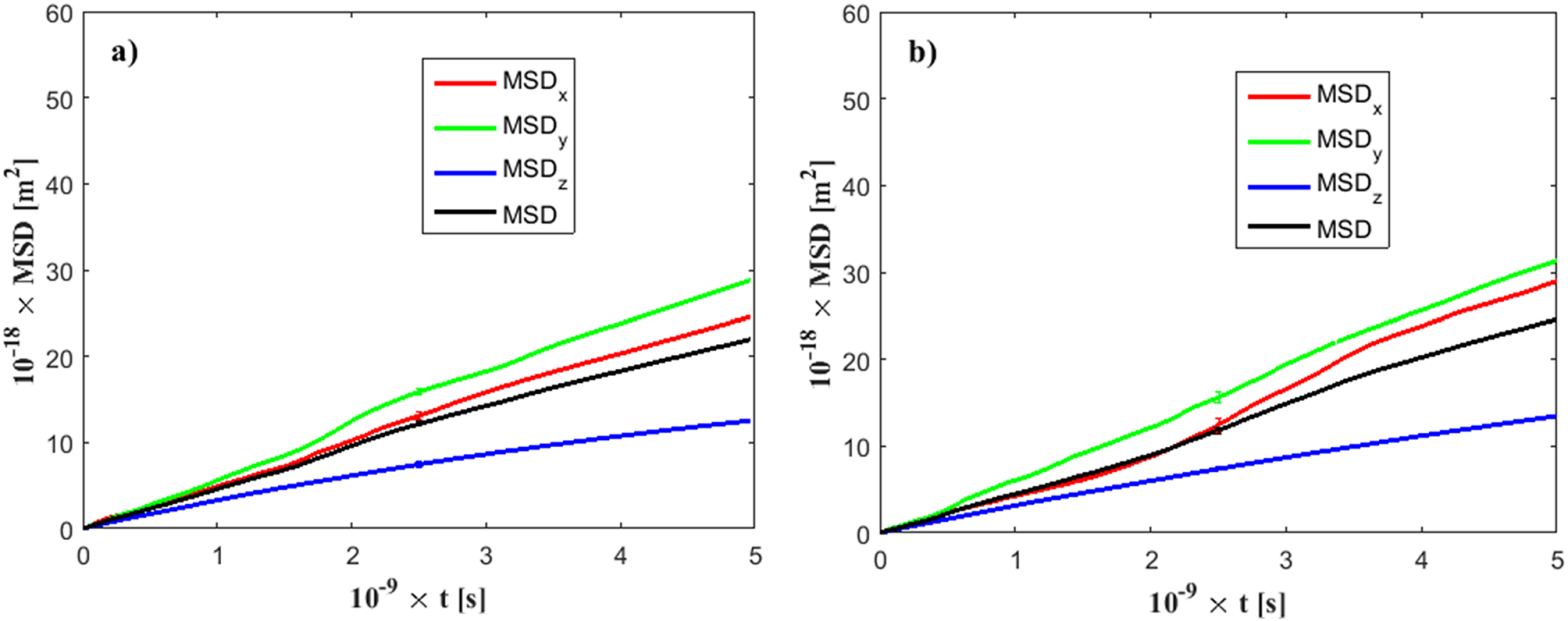
Calculated mean square displacement of fluid particles. *E*_*z*_ = 0.1 V/nm, $\varepsilon_{wf} /\varepsilon_{ff } = 0.1$. (**a**) *h* = 15 nm, (**b**) *h* = 21 nm.

To account for larger channels, we present MSD calculations for *h* = 15 nm in Fig. 5a. The effect of confinement is smaller in this case (compared to the *h* = 3 nm channel) and it has been shown that at this height range diffusion coefficients approach bulk behavior^55^. The MSD_z_ component has increased, but, still, anisotropy exists, as it is significantly smaller than MSD_x_ and MSD_y_. Similar results are obtained for the *h* = 21 nm channel, in Fig. 5b. Therefore, anisotropy tends to decrease as the channel width increases. However, electric forces and wall/fluid interaction are also sources of anisotropy^56^, even in wider nanochannels. Fluid particle diffusion parallel to *E*_*z*_ is smaller compared to the perpendicular dimension^32^, while larger diffusion values have been observed past a hydrophobic wall compared to a hydrophilic one^57^.

**Figure 6:**
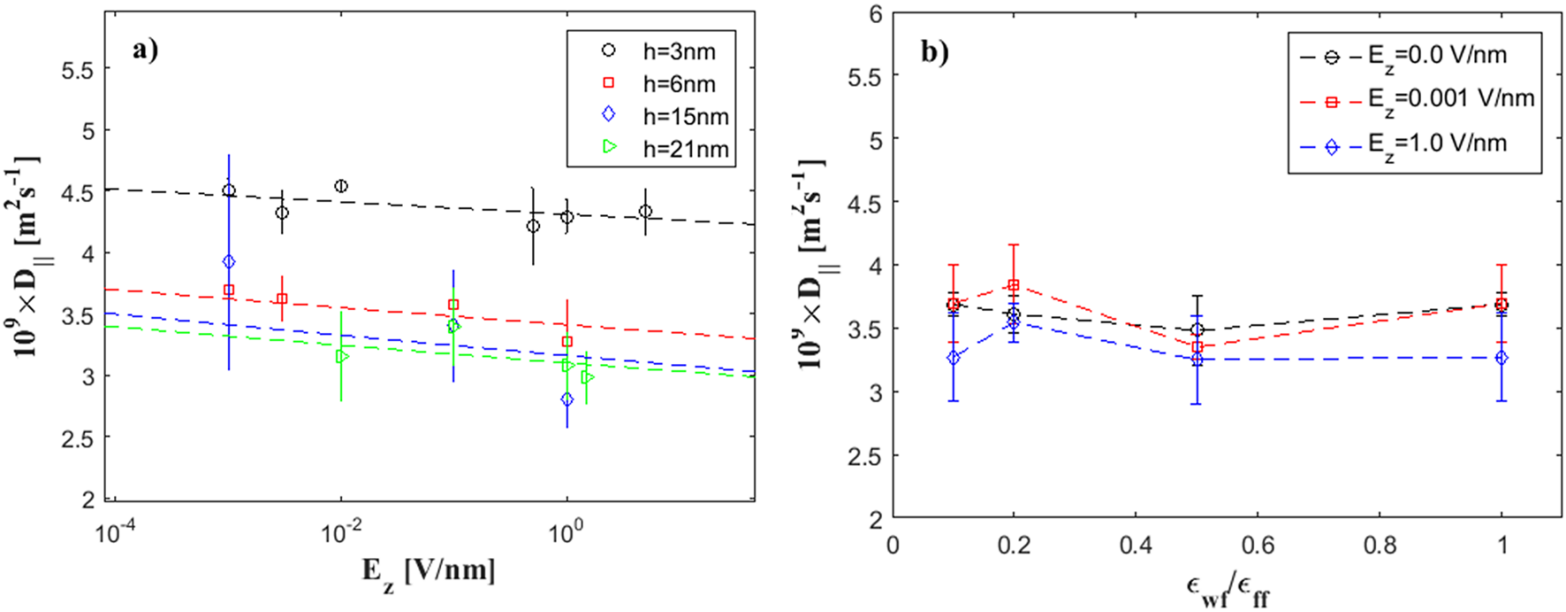
Diffusion coefficients (the component parallel to the walls) according to Eq. (9) versus (**a**) *E*_*z*_. Lines are power-law fits, (**b**) $\varepsilon_{wf} /\varepsilon_{ff }$, for the *h* = 6 nm channel. Lines are guide to the eye.

For pure water at *T* = 300 K, the bulk diffusion coefficient value ranges from *D* = 2.19 to 2.30 10^−9^m^2^s^−1^^58^. From our calculations, we have found that confinement effects impose strong anisotropy, since *D*_*z*_ values are always smaller compared to *D*_*x*_ and *D*_*y*_. In Fig. 6 we present the parallel diffusion component, *D*_*||*_, versus the external electric field and the wall wettability ratio.

Figure 6a presents *D*_*||*_ versus *E*_*z*_, for various channel heights in the range *h* = 3–21 nm. Error bars include the standard deviation of the independent simulations. We observe that the data points for every channel are well organized in power-law curves for each value of the electric field. A power-law least squares approximation of the form $D_{||} = aE_{z}^{b}$ represents the data very well. The values of the coefficients *a* and *b* are summarized in Table 1. Diffusion coefficients for all channels investigated here have a decreasing tendency as the external electric field increases. Moreover, in contrast to what expected, diffusion coefficient values are smaller in wider channels and larger in narrower channels. However, we have to bear in mind that diffusion coefficients shown here are the parallel components (over *x*- and *y*-directions).

**Table 1:** Parameters of power-law least square fits applied to Fig. 6a data.

h	a	b
3	4.31	− 0.0077
6	3.41	− 0.0087
15	3.16	− 0.0111
21	3.10	− 0.0098

In Fig. 6b the effect of wall hydrophobicity/hydrophilicity is examined, over the range $0.1 \le \varepsilon_{wf} /\varepsilon_{ff} \le 1.0$. Lines here are only for visibility reasons. It is observed that wall wettability does not have a significant effect on the parallel to the wall diffusion component, as *D*_*||*_ values for every case fluctuate around a constant value.

## Discussion

Molecular dynamics simulations have revealed many interesting characteristics of water/ion flows at the nanoscale. For desalination reasons, the model presented here is based on ion transport towards the walls, under the effect of an external electric field, perpendicular to the walls. Wall/fluid interaction strength has been taken into account to argue on its effect on ion removal.

Potential energy calculations have been presented for various conditions. Regions of low (negative) potential energy have depicted probable ion locations inside the channels. Ions could be trapped inside these positions, until an electric field of a certain value leads them towards the walls. Without an external field applied on the channel, ions can be found everywhere inside the channel. When the electric field applies, regions near the walls turn to candidate ion location positions. For weaker fields in wider nanochannels, e.g., for *E*_*z*_ = 0.001 V/nm in a *h* = 21 nm channel, ions have not obtained directional movement, they still transport in a random way and are mainly gathered near the center of the channel, as negative potential regions are formed both near the channel center and the walls, attracting fluid particles. At these cases, the proposed desalination procedure is difficult to succeed and increasing the electric field strength, though in impractical values, is the only solution.

However, ion removal is facilitated for *E*_*z*_ > 0.1 V/nm in most cases. It has been shown that, since Na^+^ and Cl^−^ ions form ionic clusters, heavier and more difficult to move, a stronger electric force is needed to overcome this barrier. It has been shown that when the electric field applies, the preferred orientation of hydrogen bonds parallel to *E* has an increasing effect on fluid viscosity, thus, hindering ion transport in *z*-dimension^33^. However, at higher values of *E*_*z*_ (e.g., for *E*_*z*_ = 1.0 V/nm), the electric field value overcomes the energy barrier and facilitates ion separation inside the nanochannel. In the water/ion solution, water molecules stick to an ion due to electrostatic interactions between them, forming a hydration shell. It is preferable for an ion to preserve this hydration shell^59^. On the other hand, a strong electric field facilitates ion drift to the walls and this has been related to the loss of the hydration shell^60^. Therefore, ions prefer to be hydrated and remain far from the wall, unless *E* is so strong that electrostatic interactions overcome the induced barrier.

Although beyond the scope of this manuscript, in chemical ion selectivity techniques, it has been found that large anions are less hydrated and are easier to remove from water solution^61^. Moreover, the different density distributions of Na^+^ and Cl^−^ ions within the pores are related to size effects, as Cl^−^ ions are heavier^62^. Extrapolating in our system, we would expect that Cl^−^ ions are easier to be directed near the walls comparing to smaller Na^+^ ions. This has been verified in our model. In a successful desalination procedure, the larger Cl^−^ ions have been directed towards the lower wall^38^, drifting smaller Na^+^ ions with them. This is why we observe ion clusters of Na^+^ and Cl^−^ together drifted towards the lower wall, while, according to the electric field orientation, only Cl^−^ ions could be found and Na^+^ ions would transport to the upper wall. This is a nanoscale effect, and it has also been observed in similar works. For example, ion density profiles have revealed neighboring layers of ions/counterions being gathered near the positive-charged graphene wall due to electrostatic attractions between them^60^.

Further calculation of the local electric field strength inside the channel, in a 3D grid form, has shown that the field lines created by inter-ion interactions can move fluid particles randomly inside the channel. For stronger *E* fields, the electric field lines are oriented parallel to the employed external field, perpendicular to the walls direction, thus forcing ions to move, and, as a result, achieve better desalination. Another parameter affecting ion drift that depends on the application is ion concentration. For the cases studied in this work, it has been found that the model achieves ion separation for a range of *c* = 0.58–1.84 M, with the same electric field strength. This is important since there is no need to increase the applied electric field when dealing with a higher concentration solution.

A means of enhancing ion drift, with reduced power consumption, could be the controlled wall hydrophobicity. However, in the channel range investigated here, *h* = 3–21 nm, it has been found that the effect of wall wettability on ionic separation from water solutions is not vital for the proposed desalination model, as it is for systems size around 1nm^29,31^. Ion transport is conquered by the electric field and wall wettability has only a small effect on it. This is also contradicting to relevant experimental results on water in capillaries^63,64^. We attribute this behavior to the presence of the external electric field, which may cause redistribution of hydrogen bonds and alter viscosity behavior^33^, which has been found to reach a higher than bulk value upon the application of an electric field^65^.

The value of the diffusion coefficient inside a channel is decreasing as the dielectric friction increases. The value of the dielectric friction is analog to the number of ion charges in the solution^66^. The formation of ion clusters inside the channels are also expected to affect diffusion coefficients.

In smaller channels (e.g., *h* = 3 nm) strong confinement effects impose anisotropy in *D* values, with *D*_*z*_ component being significantly smaller compared to *D*_*x*_ and *D*_*y*_. Calculated values of the parallel to the wall component, *D*_*||*_, decrease as the electric field increases. This can be attributed to the fact that, as Na^+^ and Cl^−^ ions are drifting towards the walls, they also drag water molecules into their hydrated shells, and this has a primitive effect on total *D*, as has been also noticed^53^.

For larger channels, the diffusion coefficient has the tendency to approach the bulk water value. However, the presence of ions and the applied electric field suggest that non-linear phenomena might happen. Wall wettability has a barely noticeable effect on *D* values.

## Conclusions

The present paper reveals that the ion behavior mechanism inside water solutions is multifactorial. Molecular Dynamics simulations have shown that the electric field strength, for values *E* = 0.0–1.0 V/nm, wall wettability strength, from strongly hydrophobic to strongly hydrophilic, ion concentration, from *c*_*ion*_ = 0.58–1.84 M, and the channel height, which spans over a range of *h* = 3–21 nm, are of major importance. Ion presence inside the channel, along with water molecules, form ion clusters and/or hydrated ions and ion separation towards the walls is hindered. For higher electric field strength values, there exists clear ion drift to the walls, as internal energy barriers are overcome. The ratio of wall/fluid interaction may be significant only in smaller nanochannels, but, in general, it does not affect ion separation at the scale investigated here. As long as the diffusion coefficients are concerned, calculated values over a wide range of system conditions have been shown. Diffusion transport presents strong anisotropy in confined channels (smaller component in the *z*-dimension compared to *x*- and *y*-), and this is pronounced in smaller electric field strengths.

To elevate the proposed simulation scheme, a future challenge would be the nano- or micro-fabrication of the proposed architecture.

## Supplementary Information

Supplementary Information.

## Data Availability

Data and codes that support the findings of this study are available from the corresponding author upon reasonable request.

## References

[1] Gogotsi Y (2018). Moving ions confined between graphene sheets. Nat. Nanotechnol..

[2] Siria A (2013). Giant osmotic energy conversion measured in a single transmembrane boron nitride nanotube. Nat..

[3] Chinappi M, Cecconi F (2018). Protein sequencing via nanopore based devices: a nanofluidics perspective. J. Phys. Condens. Matter.

[4] Shao C, Zhao Y, Qu L (2020). Tunable graphene systems for water desalination. ChemNanoMat.

[5] Abdullah N, Yusof N, Ismail AF, Lau WJ (2021). Insights into metal–organic frameworks-integrated membranes for desalination process: A review. Desalination.

[6] Guan K, Jia Y, Lin Y, Wang S, Matsuyama H (2021). Chemically converted graphene nanosheets for the construction of ion-exclusion nanochannel membranes. Nano Lett..

[7] Presumido PH, Primo A, Vilar VJP, Garcia H (2021). Large area continuous multilayer graphene membrane for water desalination. Chem. Eng. J..

[8] Wang R (2020). Recent advances in applications of carbon nanotubes for desalination: A review. Nanomaterials.

[9] Hinds BJ (2004). Aligned multiwalled carbon nanotube membranes. Science.

[10] Agrawal KV, Shimizu S, Drahushuk LW, Kilcoyne D, Strano MS (2016). Observation of extreme phase transition temperatures of water confined inside isolated carbon nanotubes. Nat. Nanotechnol..

[11] Muñoz-Santiburcio D, Marx D (2021). Confinement-controlled aqueous chemistry within nanometric slit pores. Chem. Rev..

[12] Abraham J (2017). Tunable sieving of ions using graphene oxide membranes. Nat. Nanotechnol..

[13] Padmavathy N, Behera SS, Pathan S, Das Ghosh L, Bose S (2019). Interlocked graphene oxide provides narrow channels for effective water desalination through forward osmosis. ACS Appl. Mater. Interfaces.

[14] Yang T, Lin H, Loh KP, Jia B (2019). Fundamental transport mechanisms and advancements of graphene oxide membranes for molecular separation. Chem. Mater..

[15] Landon J, Gao X, Omosebi A, Liu K (2019). Progress and outlook for capacitive deionization technology. Curr. Opin. Chem. Eng..

[16] Yang F (2021). Flow-electrode capacitive deionization: a review and new perspectives. Water Res..

[17] Zhao Y, Huang D, Su J, Gao S (2020). Coupled transport of water and ions through graphene nanochannels. J. Phys. Chem. C.

[18] Deng D (2015). Water purification by shock electrodialysis: Deionization, filtration, separation, and disinfection. Desalination.

[19] Nordstrand J, Dutta J (2021). Design principles for enhanced up-scaling of flow-through capacitive deionization for water desalination. Desalination.

[20] Atomic-scale ion transistor with ultrahigh diffusivity. https://www.science.org/doi/10.1126/science.abb5144. 10.1126/science.abb5144 33926952

[21] de Freitas DN (2020). Water diffusion in carbon nanotubes under directional electric frields: coupling between mobility and hydrogen bonding. Chem. Phys..

[22] Keerthi A (2021). Water friction in nanofluidic channels made from two-dimensional crystals. Nat. Commun..

[23] Chen L, Wang SY, Xiang X, Tao WQ (2020). Mechanism of surface nanostructure changing wettability: a molecular dynamics simulation. Comput. Mater. Sci..

[24] Mahmood A (2020). Spontaneous propulsion of a water nanodroplet induced by a wettability gradient: A molecular dynamics simulation study. Phys. Chem. Chem. Phys..

[25] Ranathunga DTS, Shamir A, Dai X, Nielsen SO (2020). Molecular dynamics simulations of water condensation on surfaces with tunable wettability. Langmuir.

[26] De Luca S, Todd BD, Hansen JS, Daivis PJ (2013). Electropumping of water with rotating electric fields. J. Chem. Phys..

[27] Chen Q (2011). Water transport and purification in nanochannels controlled by asymmetric wettability. Small.

[28] Zong D, Yang Z, Duan Y (2017). Wettability of a nano-droplet in an electric field: a molecular dynamics study. Appl. Therm. Eng..

[29] Klesse G, Tucker SJ, Sansom MSP (2020). Electric Field induced wetting of a hydrophobic gate in a model nanopore based on the 5-HT3Receptor channel. ACS Nano.

[30] Kamal Kandezi M, Shadman Lakmehsari M, Matta CF (2020). Electric field assisted desalination of water using B- and N-doped-graphene sheets: A non-equilibrium molecular dynamics study. J. Mol. Liq..

[31] Lynch CI, Rao S, Sansom MSP (2020). Water in nanopores and biological channels: a molecular simulation perspective. Chem. Rev..

[32] Shafiei M, Von Domaros M, Bratko D, Luzar A (2019). Anisotropic structure and dynamics of water under static electric fields. J. Chem. Phys..

[33] Zong D, Hu H, Duan Y, Sun Y (2016). Viscosity of water under electric field: anisotropy induced by redistribution of hydrogen bonds. J. Phys. Chem. B.

[34] Bruus H (2008). Theoretical microfluidics.

[35] Song C, Corry B (2011). Testing the applicability of Nernst-Planck theory in ion channels: comparisons with brownian dynamics simulations. PLoS ONE.

[36] Bartzis V, Sarris IE (2021). Time evolution study of the electric field distribution and charge density due to ion movement in salty water. Water.

[37] Bartzis V, Sarris IE (2020). A theoretical model for salt ion drift due to electric field suitable to seawater desalination. Desalination.

[38] Sofos F, Karakasidis T, Sarris IE (2020). Molecular dynamics simulations of ion drift in nanochannel water flow. Nanomaterials.

[39] Jorgensen WL, Chandrasekhar J, Madura JD, Impey RW, Klein ML (1983). Comparison of simple potential functions for simulating liquid water. J. Chem. Phys..

[40] Plimpton S (1995). Fast parallel algorithms for short-range molecular dynamics. J. Comput. Phys..

[41] Voronov RS, Papavassiliou DV, Lee LL (2006). Boundary slip and wetting properties of interfaces: Correlation of the contact angle with the slip length. J. Chem. Phys..

[42] Sofos F, Karakasidis TE, Liakopoulos A (2012). Surface wettability effects on flow in rough wall nanochannels. Microfluid. Nanofluidics.

[43] Jiang H, Müller-Plathe F, Panagiotopoulos AZ (2017). Contact angles from Young’s equation in molecular dynamics simulations. J. Chem. Phys..

[44] Saitta AM, Saija F, Giaquinta PV (2012). Ab initio molecular dynamics study of dissociation of water under an electric field. Phys. Rev. Lett..

[45] Moqadam M (2018). Local initiation conditions for water autoionization. Proc. Natl. Acad. Sci..

[46] Qiu H, Guo W (2013). Electromelting of confined monolayer ice. Phys. Rev. Lett..

[47] Aksimentiev A, Schulten K (2005). Imaging α-hemolysin with molecular dynamics: ionic conductance, osmotic permeability, and the electrostatic potential map. Biophys. J..

[48] Sega M, Sbragaglia M, Biferale L, Succi S (2015). The importance of chemical potential in the determination of water slip in nanochannels. Eur. Phys. J. E.

[49] Huang DM, Sendner C, Horinek D, Netz RR, Bocquet L (2008). Water slippage versus contact angle: a quasiuniversal relationship. Phys. Rev. Lett..

[50] Ritos K, Mattia D, Calabrò F, Reese JM (2014). Flow enhancement in nanotubes of different materials and lengths. J. Chem. Phys..

[51] Large-scale, robust mushroom-shaped nanochannel array membrane for ultrahigh osmotic energy conversion. https://www.science.org/doi/10.1126/sciadv.abg2183. 10.1126/sciadv.abg2183 PMC8133705 34138731

[52] Hu H, Bao L, Priezjev NV, Luo K (2017). Identifying two regimes of slip of simple fluids over smooth surfaces with weak and strong wall-fluid interaction energies. J. Chem. Phys..

[53] Li JH (2020). Insights on the ion migration throughout the nano-channel of ettringite under an external electric field: structure, dynamics, and mechanisms. Constr. Build. Mater..

[54] Esfandiar A (2017). Size effect in ion transport through angstrom-scale slits. Science.

[55] Sofos F, Karakasidis T, Liakopoulos A (2009). Transport properties of liquid argon in krypton nanochannels: Anisotropy and non-homogeneity introduced by the solid walls. Int. J. Heat Mass Transf..

[56] Crozier PS, Rowley RL, Henderson D (2000). Molecular dynamics calculations of the electrochemical properties of electrolyte systems between charged electrodes. J. Chem. Phys..

[57] Sofos F, Karakasidis TE, Giannakopoulos AE, Liakopoulos A (2016). Molecular dynamics simulation on flows in nano-ribbed and nano-grooved channels. Heat Mass TransferWaerme- Stoffuebertragung.

[58] Harris KR, Woolf LA (1980). Pressure and temperature dependence of the self diffusion coefficient of water and oxygen-18 water. J. Chem. Soc. Faraday Trans. 1 Phys. Chem. Condens. Phases.

[59] Qiao R, Aluru NR (2005). Atomistic simulation of KCl transport in charged silicon nanochannels: interfacial effects. Colloids Surf. Physicochem. Eng. Asp..

[60] Kalluri RK, Konatham D, Striolo A (2011). Aqueous NaCl solutions within charged carbon-slit pores: Partition coefficients and density distributions from molecular dynamics simulations. J. Phys. Chem. C.

[61] Liu Y, Zhao W, Chen CH, Flood AH (2019). Chloride capture using a C–H hydrogen-bonding cage. Science.

[62] Argyris D, Cole DR, Striolo A (2010). Ion-specific effects under confinement: the role of interfacial water. ACS Nano.

[63] Gruener S, Hofmann T, Wallacher D, Kityk AV, Huber P (2009). Capillary rise of water in hydrophilic nanopores. Phys. Rev. E Stat. Nonlinear Soft Matter Phys..

[64] Malfait B (2021). Influence of pore surface chemistry on the rotational dynamics of nanoconfined water. J. Phys. Chem. C.

[65] Kimura H, Sugiyama T, Takahashi S, Tsuchida A (2013). Viscosity change in aqueous hectorite suspension activated by DC electric field. Rheol. Acta.

[66] Samanta T, Matyushov DV (2020). Mobility of large ions in water. J. Chem. Phys..

