# Supplementary material for: Effects of channel size, wall wettability, and electric field strength on ion removal from water in nanochannels
Source: Sci Rep. 2022 Jan 12;12:641. doi: 10.1038/s41598-021-04620-x (PMC8755770; doi:10.1038/s41598-021-04620-x)
Supplement: Supplementary file 1 — Supplementary Information. [file 41598_2021_4620_MOESM1_ESM.docx]

Effects of channel size, wall wettability, and electric field strength on ion removal from water in nanochannels

Filippos Sofos^1, 🖂^, Theodoros E. Karakasidis^1^, Ioannis E. Sarris^2^

^1^ Condensed Matter Physics Laboratory, Department of Physics, University of Thessaly, 35100 Lamia, Greece

^2^ Department of Mechanical Engineering, University of West Attica, 12244 Athens, Greece

***** e-mail: fsofos@uth.gr

**Supplementary material**

**Simulation setup****.** Parameters of the simulation are presented in Table S1, and interaction parameters between atoms in Table S2 ^S1^. Arithmetic Lorentz–Berthelot combining rules are applied to all interatomic interactions according to Table S2 values, i.e., for two different species *α* and *b* we consider $\sigma_{ab}=\left( \sigma_{a}+\sigma_{b} \right)/2$, $\varepsilon_{ab}=\sqrt{\left( \varepsilon_{a}\varepsilon_{b} \right)}$.

The simulation procedure is shown graphically in Figure S1. All snapshots have been produced with OVITO ^S2^. At first (Figure S1a) the initial placement is shown, with ion concentration c=0.58M. Wall atoms remain frozen with fcc structure. Next, (Figure S1b) a NPT simulation follows and fluid particles attain their new positions and velocities in the box. Box dimensions are allowed to change at this step, to ensure constant pressure (*P*=3000atm) and temperature (*T*=300K). As the z-direction is periodic, the adjustments are performed in the x- and y-directions. Another 1 ns of NVE simulation follows, and after that, a NVT scheme with thermostats at the walls, where the electric field is applied for 5 ns. This simulation time, as shown from Figure S1c, is adequate for ions to drift towards the walls. At this point, production runs start (NVT at the walls - NVE for the fluid) and simulation parameters are saved for our calculations (Figure S1d), for total time of 20 ns (4 consecutive, independent runs of 5 ns each).

With increased ion concentration (c=0.92M), simulation snapshots of Figure S2 are obtained. Moreover, for weaker external electric field (*E*=0.1V/nm), Figures S3 are obtained for various channel heights.

**Table S1.** Simulation parameters. Number of ions shown correspond to *c*=0.58M.

| *Channel height, h* (nm) | 3 | 6 | 9 | 15 | 21 |
| --- | --- | --- | --- | --- | --- |
| *Computational dimension in x, L_x_ (nm)* | 3.32 | 2.92 | 3.01 | 2.86 | 3.0 |
| *Computational dimension in y, L_y_ (nm)* | 1.56 | 1.51 | 1.57 | 1.56 | 1.54 |
| *Computational dimension in z, L_z_ (nm)* | 3.72 | 7.44 | 10.23 | 18.6 | 23.72 |
| *Division in z-bins, m* | 37 | 74 | 102 | 186 | 237 |
| *Division in x-bins, n* | 31 | | | | |
| *Division in y-bins, k* | 1 | | | | |
| *Number of water molecules, N_H2O_* | 500 | 1000 | 1500 | 2500 | 3750 |
| *Number of Na^+^, N_Na+_* | 5 | 10 | 15 | 25 | 35 |
| *Number of Cl^-^, N_Cl-_* | 5 | 10 | 15 | 25 | 35 |
| *Number of wall atoms, N_w_* | 342 | | | | |
| *Ion concentration, c_ion_ (M)* | 0.58, 0.92, 1.38, 1.84 | | | | |
| *External electric field, E_z_* (V/nm) | 0.0 – 10.0 | | | | |
| *Wall-to-fluid ε ratio, εwf/εff* | 0.1 – 1.0 | | | | |
| *Temperature, T (K)* | 300 | | | | |
| *Pressure, (atm)* | 3000 | | | | |

**Table S2.** Parameters (*ε*, *σ*, and *m*) for each atom in the solution. C corresponds to the LJ wall atom, with parameters equal to carbon. Interaction O-C denotes wall wettability and ranges from 0.01852-0.1852 Kcal/mol.

| Atom | ε (Kcal/mol) | σ(Å) | mass (a.u.) |
| --- | --- | --- | --- |
| H-H | 0.000 | 0.000 | 1.008 |
| O-O | 0.155 | 3.166 | 15.99 |
| C-C | 0.056 | 3.400 | 12.01 |
| Cl-Cl | 0.107 | 4.446 | 35.45 |
| Na-Na | 1.607 | 1.897 | 22.99 |
| H-O | 0.000 | 0.000 | - |
| H-C | 0.000 | 0.000 | - |
| H-Cl | 0.000 | 0.000 | - |
| H-Na | 0.000 | 0.000 | - |
| O-C | 0.0185-0.185 | 3.279 | - |
| O-Cl | 0.046549 | 4.1617 | - |
| O-Na | 0.1766 | 2.7089 | - |
| C-Cl | 0.031702 | 4.2821 | - |
| C-Na | 0.12027 | 2.8293 | - |
| Cl-Na | 0.044388 | 3.7117 | - |

| **(a) 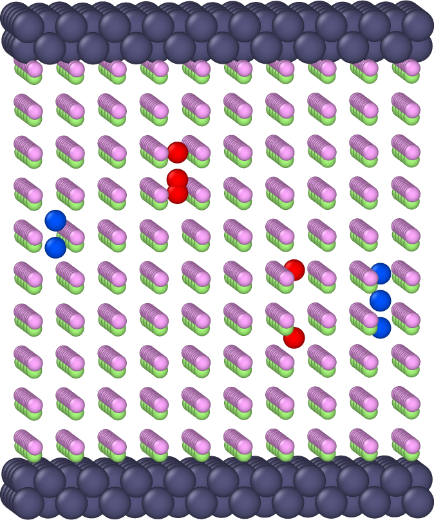** | **(b) 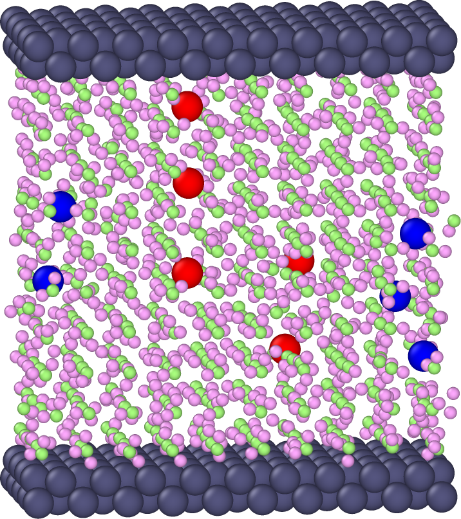** |
| --- | --- |
| **(c) 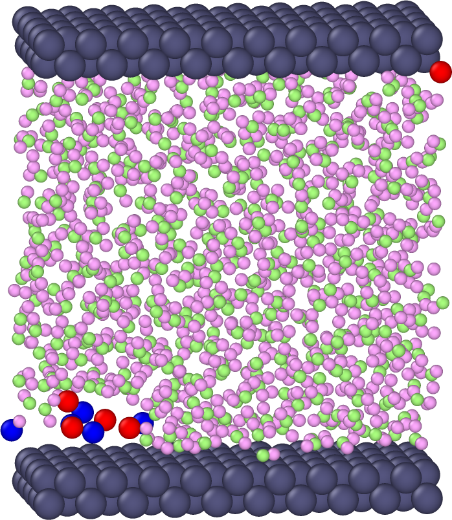** | **(d) 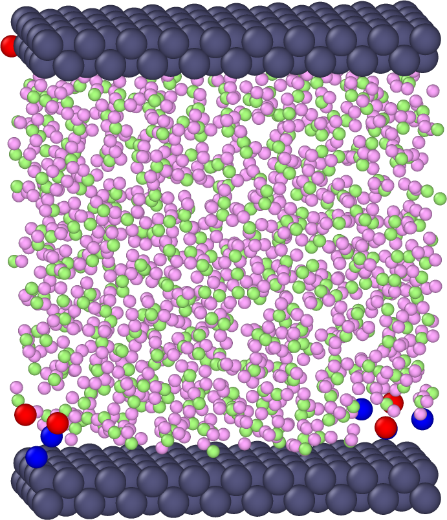** |

**Figure S1.** Simulation snapshots, $h=3nm$, ${\varepsilon_{wf}}/{\varepsilon_{ff}=0.1}$, $E_{z}=1.0 V/nm$, c=0.58M. **a)** Initial simulation setup, **b)** After the NPT equilibration, in adjusted box dimensions, **c)** The electric field applies and ions drift to the walls, **d)** Production run. Particle colors are, grey: wall, red: Na^+^, blue: Cl^-^, black: C, pink: H, green: O. Hydrogen and oxygen atoms are shown shrunk, natrium and chloride ions are magnified for visibility reasons.

| **(a) 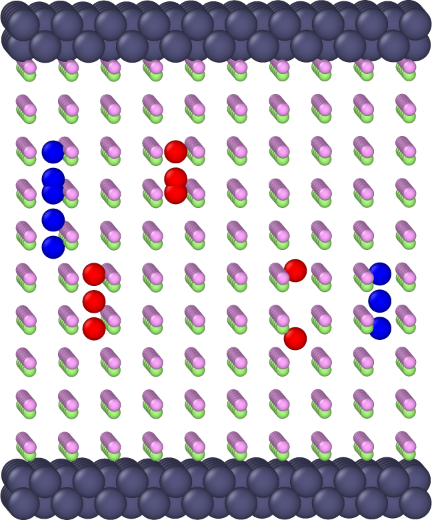** | **(b) 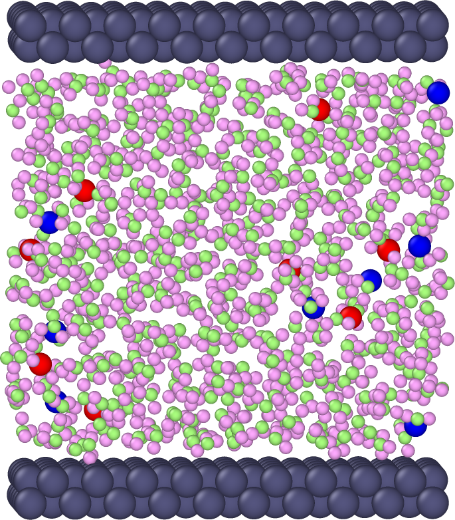** |
| --- | --- |
| **(c) 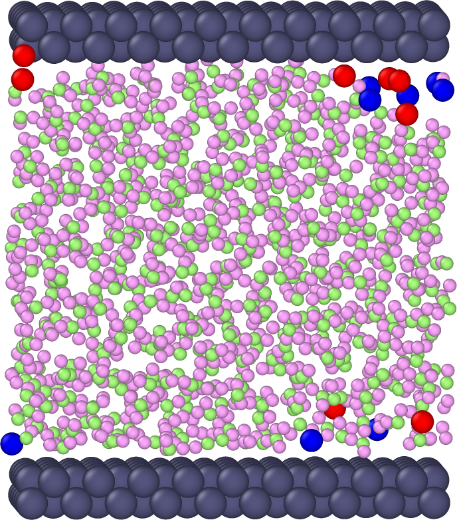** | **(d) 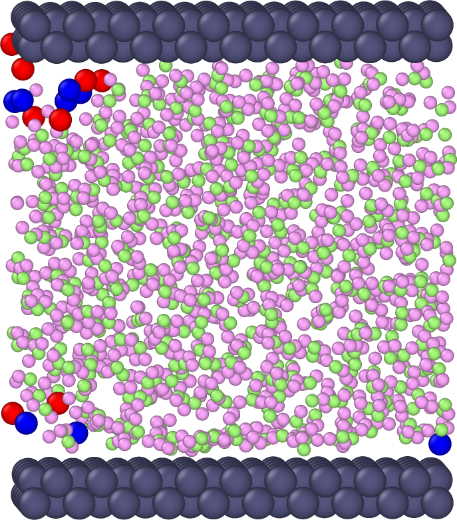** |

**Figure S2.** As in Figure S1, but for c=0.92M. Particle colors are, grey: wall, red: Na^+^, blue: Cl^-^, black: C, pink: H, green: O. Hydrogen and oxygen atoms are shown shrunk, natrium and chloride ions are magnified for visibility reasons.

| **(a) 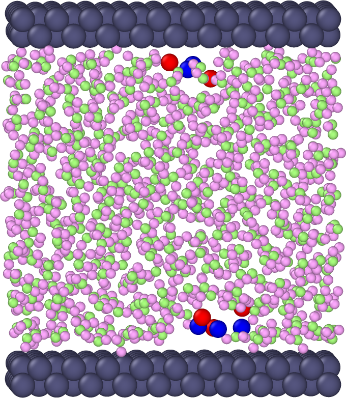** | **(b) 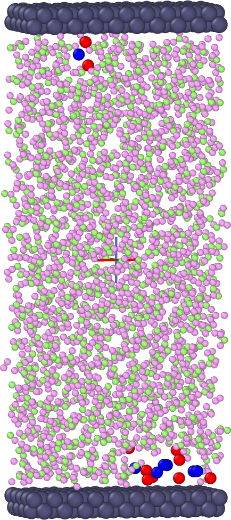** |
| --- | --- |
| **(c) 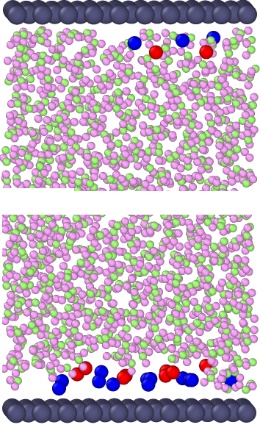** | **(d) 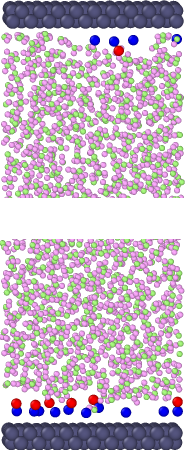** |

**Figure S3.** Simulation snapshots for $E_{z}=0.1 V/nm$, just before the production runs start. ${\varepsilon_{wf}}/{\varepsilon_{ff}=0.1}$, c=0.58M. **a)** $h=3nm,$ **b)** $h=6nm$, **c)** $h=9nm$, **d)** $h=15nm$. Particle colors are, grey: wall, red: Na^+^, blue: Cl^-^, black: C, pink: H, green: O. Hydrogen and oxygen atoms are shown shrunk, natrium and chloride ions are magnified for visibility reasons.

Four indicative pressure and temperature plots are shown in Figure S4, for *h*=3-15nm. After the initial setup, *P* and *T* are stabilized around the imposed value. The base pressure value is the equilibrium value of the *h*=3nm channel and it was selected by a previous NVT simulation.

| **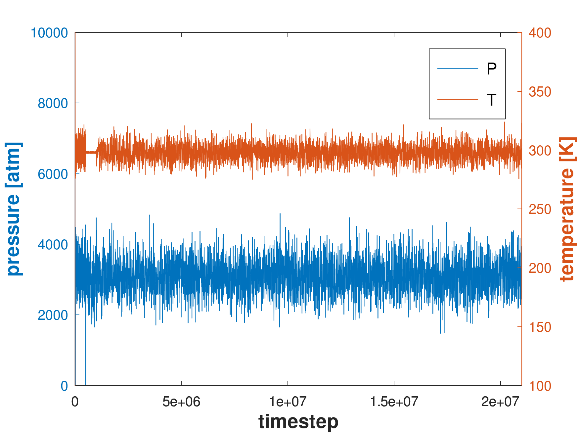(a)** | **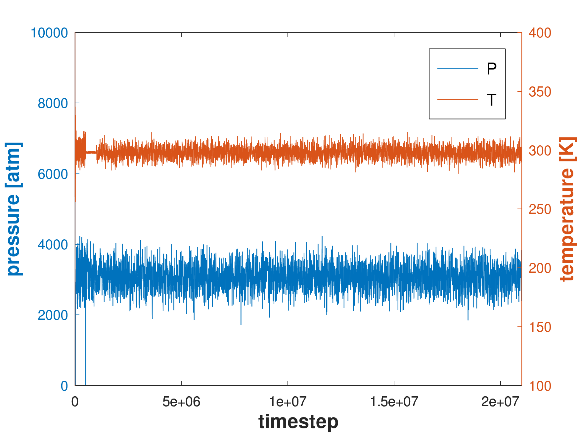(b)** |
| --- | --- |
| **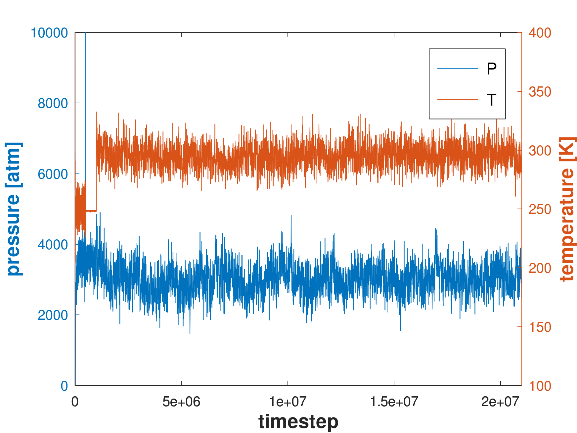(c)** | **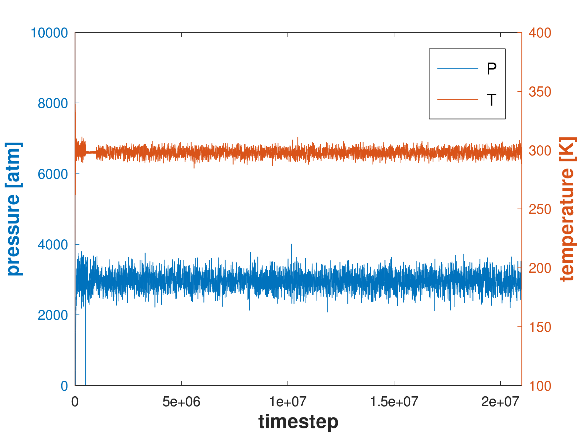(d)** |

**Figure S4.** Pressure and temperature vs. simulation timestep. The vertical axis on the left presents pressure values and the right axis the temperature. Both temperature and pressure values fluctuate around a set value controlled by the simulations. ${\varepsilon_{wf}}/{\varepsilon_{ff}=0.1}$, $E_{z}=1.0 V/nm$. **a)** $h=3nm$, **b)** $h=6nm$, **c)** $h=9nm$, **d)** $h=15nm$.

Figure S5 presents the calculated MSD for various wall wettability ratios, $0.1{{\leq\varepsilon}_{wf}}/{\varepsilon_{ff}\leq1.0}$.

| **(a)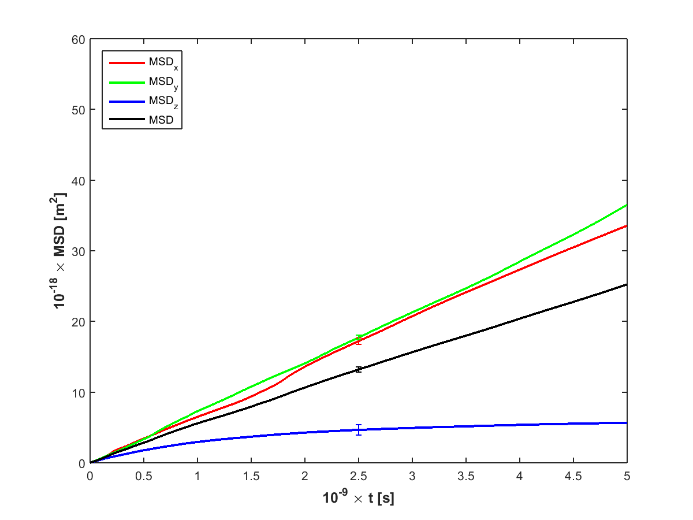** | **(b)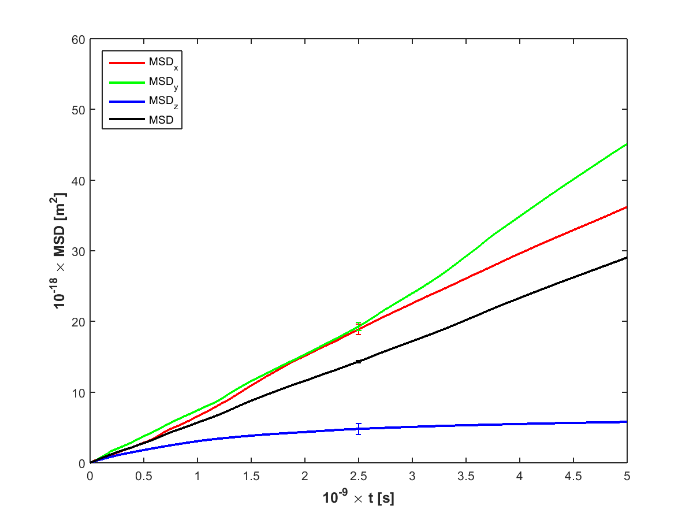** |
| --- | --- |
| **(c)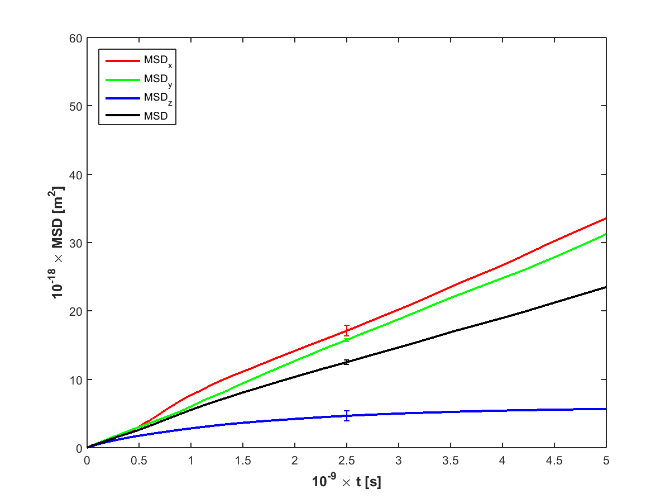** | **(d)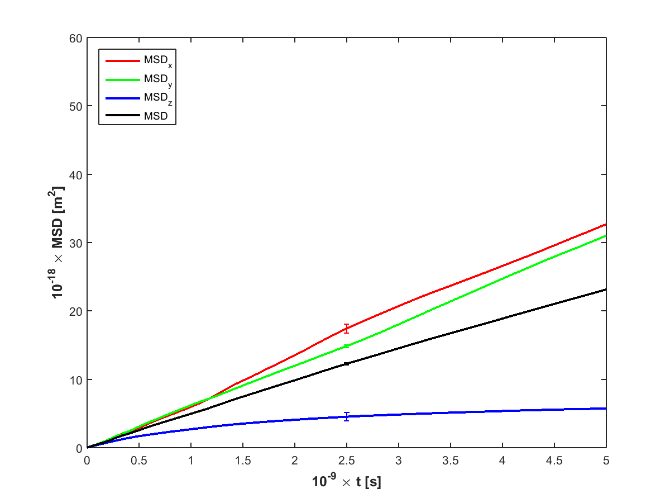** |

**Figure S5**. Calculated mean square displacement of fluid particles. *E_z_*=0.01V/nm, *h*=6nm. **a)** ${\varepsilon_{wf}}/{\varepsilon_{ff}=0.1}$, **b)** ${\varepsilon_{wf}}/{\varepsilon_{ff}=0.2}$, **c)** ${\varepsilon_{wf}}/{\varepsilon_{ff}=0.5}$, **d)** ${\varepsilon_{wf}}/{\varepsilon_{ff}=1.0}$.

**References**

1. Cohen-Tanugi, D. & Grossman, J.C. Water permeability of nanoporous graphene at realistic pressures for reverse osmosis desalination. *J. Chem. Phys.* **141**, 074704 (2014).
2. Stukowski, A. Visualization and analysis of atomistic simulation data with OVITO - the Open Visualization Tool. *Model. Simul. Mat. Sci. Eng.* **18**, 015012 (2010).
